# When survival estimates mask underlying realities: Interpreting global prostate cancer survival disparities from registry data

**DOI:** 10.1016/j.jncc.2026.07.003

**Published:** 2026-08-11

**Authors:** Xuefan Zeng

**Affiliations:** Chongqing Medical University, Chongqing, China

We read with great interest Zhou et al.’s comprehensive global article of prostate cancer survival.^1^ The authors revealed that while survival had improved, substantial disparities persist—ranging from 98.5% in the United States to 30.3% in India. However, caution is warranted when drawing inferences and policy recommendations, given the methodological limitations, profound confounding by prostate-specific antigen (PSA) screening, and differential registry data quality.

First, although the authors acknowledge that “survival must be interpreted cautiously due to lead-time bias”, this critical limitation deserves far more prominent consideration than it receives in the article. The widespread adoption of PSA screening in Western countries since the late 1980s has fundamentally altered the epidemiology of prostate cancer in ways that render simple survival comparisons between screened and unscreened populations deeply problematic. Lead time—the interval by which screening advances diagnosis—averages 5 to 7 years for prostate cancer,^2^ meaning that a substantial proportion of the apparent survival advantage in high-income countries may reflect earlier diagnosis rather than genuine improvements in prognosis. Indeed, modeling studies have demonstrated that lead time and overdiagnosis explain nearly all of the apparent survival improvements at older ages, with real survival benefits in the PSA era ranging from only 25% to 32% after accounting for these artifacts.^3^ The authors’ observation that the United States has maintained 5-year relative survival approaching 100% since the early 2000s must be interpreted in this context: such figures may reflect the detection of a large reservoir of indolent, clinically insignificant tumors rather than meaningful progress against lethal disease.

Second, the heterogeneity in data quality and registry completeness across countries substantially undermines the validity of cross-national comparisons. The authors appropriately note that “most studies are based on one or a few cancer registries, which may not fully represent the entire population”. However, this limitation is more severe than acknowledged. In many low- and middle-income countries, cancer registries suffer from incomplete case ascertainment, passive follow-up with delayed or failed linkage to death records, and variable coverage that may not achieve even 50% of the true incident cases.^4^ For example, data from sub-Saharan Africa—where the authors report some of the lowest survival estimates—are derived from registries that cover only select urban populations and may systematically under-ascertain deaths, leading to overestimation rather than underestimation of survival.^5^ Conversely, incomplete death registration can produce the opposite bias: if deaths are missed, survival appears artificially inflated.^6^ The lack of standardized data quality metrics across the included studies makes it nearly impossible to determine the direction or magnitude of these biases.

Third, the subgroup analysis by age and stage—while valuable—is compromised by substantial missing data and inconsistent reporting. The authors report that only 46% of cases in some registries had complete data on basic cancer characteristics for risk stratification,^7^ and the excluded cases differed significantly from those included across every reported variable. This missing data pattern is not random; patients with missing stage information are more likely to be elderly, have comorbidities, or be diagnosed in settings with limited diagnostic workup—all factors associated with poorer outcomes. The authors’ observation that 5-year relative survival for “unknown stage” cases varies widely across countries likely reflects differing proportions of early-stage patients within this category, but this heterogeneity also means that stage-specific comparisons may be biased by differential completeness of staging information across regions.

Fourth, the review’s emphasis on relative survival as the primary metric warrants methodological scrutiny. While relative survival aims to estimate cancer-specific mortality by comparing observed survival to that expected in the general population, this approach relies on the assumption that the general population mortality experience is accurately captured by life tables. In countries with incomplete vital registration systems, the expected survival estimates may be substantially biased. Moreover, relative survival does not account for competing risks in a way that is easily interpretable for clinical decision-making; a patient with prostate cancer and multiple comorbidities may have a high relative survival (because the cancer itself is not the cause of death) but poor overall prognosis. The authors acknowledge that “patients aged ≥75 had the poorest prognosis” but attribute this to “more advanced stages, limited treatment intensity, and a higher comorbidity burden”—yet the relative survival metric cannot disentangle these competing explanations.

Last, while the authors appropriately highlight socioeconomic and healthcare system factors as drivers of survival disparities, they do not adequately address the role of treatment access and affordability as distinct from healthcare system sophistication. Even within high-income countries, substantial disparities exist by insurance status and socioeconomic position. Data from Brazil, for example, demonstrate that patients in the public healthcare system have access to fewer lines of therapy for metastatic prostate cancer and significantly shorter median survival than those treated in the private system.^8^ Similarly, in the United States, county-level poverty and education are independently associated with prostate cancer mortality, with the worst poverty quintile associated with a 38% increase in mortality risk. These within-country disparities suggest that national-level averages may mask profound inequities that are at least as important as between-country differences.

To advance this work, future research should incorporate standardized data quality metrics for meaningful comparisons. First, apply period analysis rather than cohort analysis for up-to-date estimates. Second, the application of period analysis rather than cohort analysis would provide more up-to-date estimates by incorporating the survival experience of patients diagnosed in recent calendar periods.^9^ Third, the integration of socioeconomic and healthcare access data at the individual or area level would allow disentanglement of the relative contributions of screening, treatment, and healthcare system factors to observed survival disparities. Fourth, prospective studies with standardized protocols are urgently needed to validate these findings and capture patient-reported outcomes.

In conclusion, while Zhou et al. provide a crucial foundation, interpreting registry-based survival comparisons requires caution. Future research must conduct integrated analyses accounting for screening, data quality, healthcare access, and patient-centered outcomes to ensure global disparities reduction is grounded in robust evidence.

## Declaration of AI and AI-assisted technologies in the writing process

During the preparation of this manuscript, ChatGPT (OpenAI) was employed exclusively for grammatical and stylistic editing.

## Declaration of competing interest

The author declares that he has no known competing financial interests or personal relationships that could have appeared to influence the work reported in this paper.
